# A method of hollowing the obturator prosthesis and an overview on the pros and cons of the various materials used for hollowing

**DOI:** 10.25122/jml-2020-0142

**Published:** 2021

**Authors:** Sharayu Vinod Nimonkar, Vikram Murlidhar Belkhode, Ali Mohammed Asiri, Mohammed Fawaz Aldossary, Pranali Vinod Nimonkar

**Affiliations:** 1. Department of Prosthodontics, Sharad Pawar Dental College & Hospital, Datta Meghe Institute of Medical Sciences (Deemed to be University), Sawangi (Meghe), Wardha, Maharashtra, India; 2. College of Dentistry, Prince Sattam Bin Abdulaziz University, Al-Kharj, Saudi Arabia; 3. Trauma Care Centre, Government Medical College and Hospital, Nagpur, Maharashtra, India

**Keywords:** hollow bulb obturator, ice, maxillectomy

## Abstract

Prosthetic rehabilitation of a partial or total maxillectomy with an obturator is the most acceptable treatment option. The hollowing of the obturator prosthesis is beneficial as it reduces the stresses over the underlying and surrounding tissues. A simple technique of fabricating a hollow bulb obturator has been discussed in this article. At the step of the packing of a denture, the hollow wax pattern of the defect area is formed with modeling wax. This hollow wax pattern is filled with water and is allowed to freeze to form an ice block. This ice block is removed from the wax pattern and is interposed between two layers for heat-cured acrylic resin and is then cured. After processing the denture, the water is retrieved by making a small hole in denture base, which is packed after hollowing with a cold cure acrylic resin. A lightweight prosthesis with a uniform thickness was achieved with a readily available and easily retrievable material, i.e., ice.

## INTRODUCTION

Maxillectomy is a term used for the removal of the partial or total maxilla in patients with benign or malignant tumors. Rehabilitating such maxillary defects with free flap surgery has shown the best results functionally as well as esthetically. Prosthetic rehabilitation is the preferred modality only when surgical intervention is not possible [1]. Patients with advanced age, poor general health, extensive defects, and poor blood supply because of radiation therapy can be considered for prosthetic rehabilitation [2].

The hard and/or soft palate defect leading to oro-nasal or oro-antral communication often leads to physical and psychological trauma to the patient. Patients planned for maxillectomy often ask about the quality of life (QoL) that they should expect after surgery. There are many studies documented in the literature supporting a good quality of life provided by well-designed obturators prosthesis following maxillectomy [3–8].

Obturator prosthesis is a removable appliance used to close the congenital or acquired tissue opening primarily of the hard palate and/ or soft palate. This bulb of the obturator engages the defect to gain retention for the prosthesis and form a barrier for oral and nasopharyngeal communication. The bulb of the obturator prosthesis increases the weight of the prosthesis. An increase in weight hampers the retention of the prosthesis that makes the prosthesis unacceptable by the patient [9].

To avoid this, the obturator prosthesis should be made hollow. Various methods for hollowing the prosthesis with the use of different materials have been well documented in studies [10–15]. However, clinically, it has been observed that the removal of these materials from processed prostheses is a tacky job. Moreover, they could not maintain the even thickness of the hollow portion; some caused porosities, dimensional changes, and discoloration of the final prosthesis. Different techniques such as the double flask technique and use of shim of acrylic resin have been given for hollowing of the prosthesis by Fattore *et al*. [16] and Holt *et al*. [17], respectively. However, the problems faced with such techniques were an approximation of two half polymerized portions of the prosthesis and the even thickness of the prosthesis.

This article describes the technique for the fabrication of hollow obturator prosthesis using a freely accessible material, i.e., ice. A hollow wax pattern was formed by adapting modeling wax on the borders of the defect cavity. Water was filled in a hollow wax pattern to form an ice block. This ice block was placed at the time of curing as a hollowing material. After processing the denture, the ice water was retrieved by making a small hole in the denture base.

The ice block used in this method as a material for hollowing could withstand the pressure of the compression molding technique and did not cause discoloration of the denture, as seen with many materials used for hollowing. It also provided ease of retrievability. Moreover, this technique maintained the even thickness of the hollow portion and also aided in the complete curing of the bulb portion.

## TECHNIQUE

**Figure 1:**
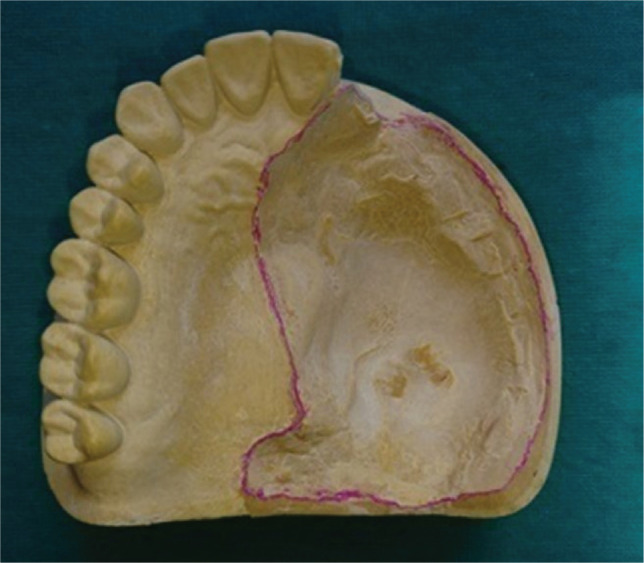
Maxillary cast showing the defect.

**Figure 2:**
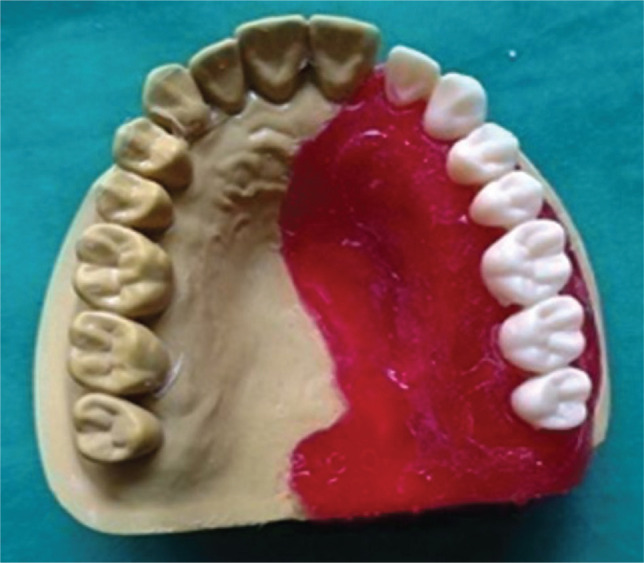
Trial prosthesis.

**Figure 3:**
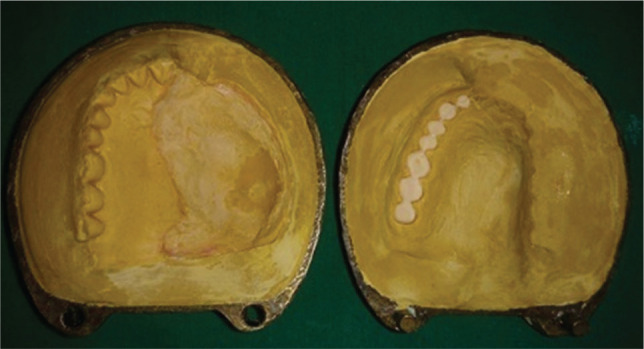
Dewaxed flask.

**Figure 4:**
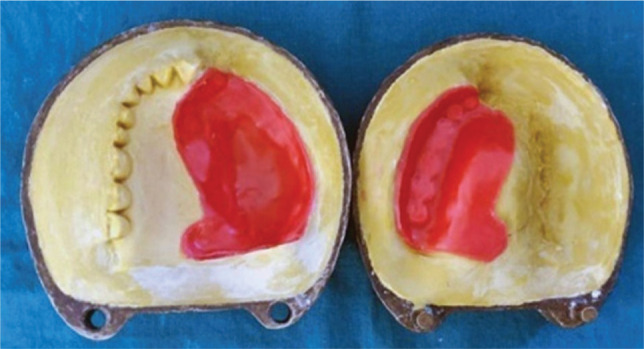
Wax sheet adapted on the defect area to form the wax pattern.

All clinical and laboratory procedures for fabricating the obturator prosthesis from preliminary impression until de-waxing of the trial denture were done conventionally (Figures 1 and 2). After de-waxing (Figure 3), modeling wax (Pyrax, Pyrax Polymar, India) was softened uniformly in warm water and was adapted on the walls of the defect area on the base flask and counter flask (Figure 4). The excess wax that extended from the defect area was flamed, and the flask was closed to get a wax pattern.

**Figure 5:**
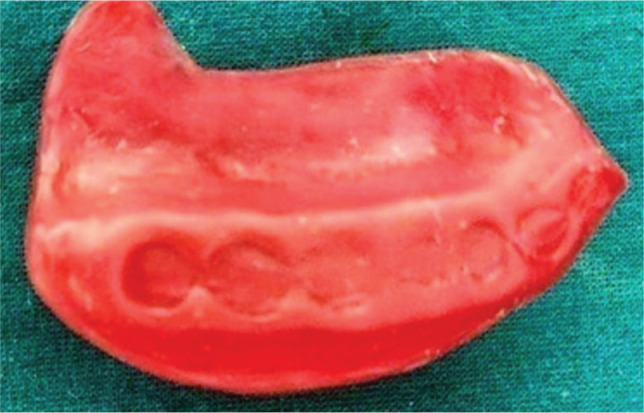
The wax pattern.

**Figure 6:**
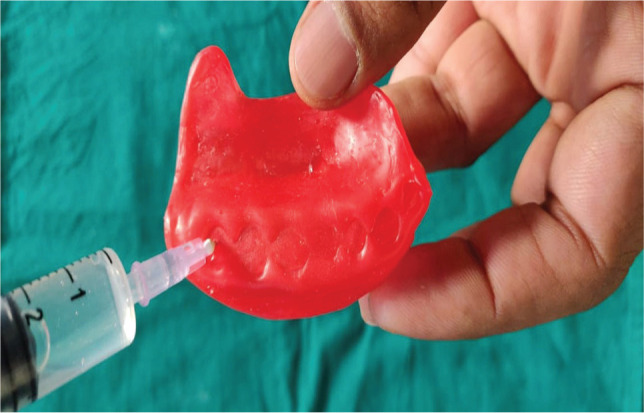
Injection of water into the wax pattern.

The wax pattern was removed carefully from the flask and was checked for the seal (Figure 5). Water was injected with the help of a syringe in the wax pattern (Figure 6). The wax pattern, along with water in it, was kept in the freezer for 24 hrs to form ice.

**Figure 7:**
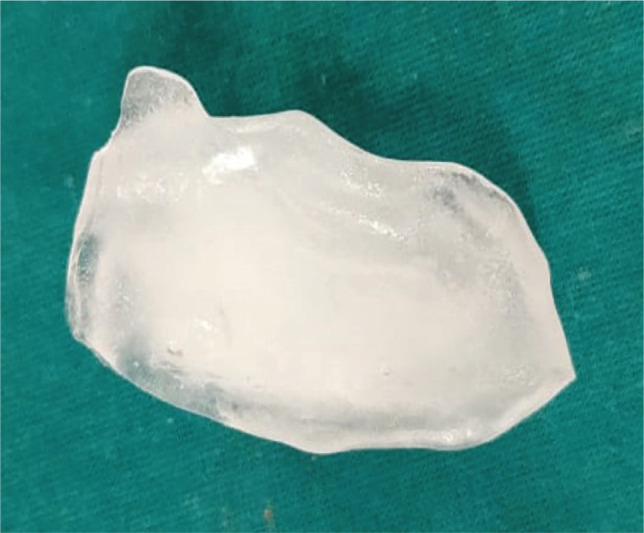
Ice block retrieved from the wax pattern.

**Figure 8:**
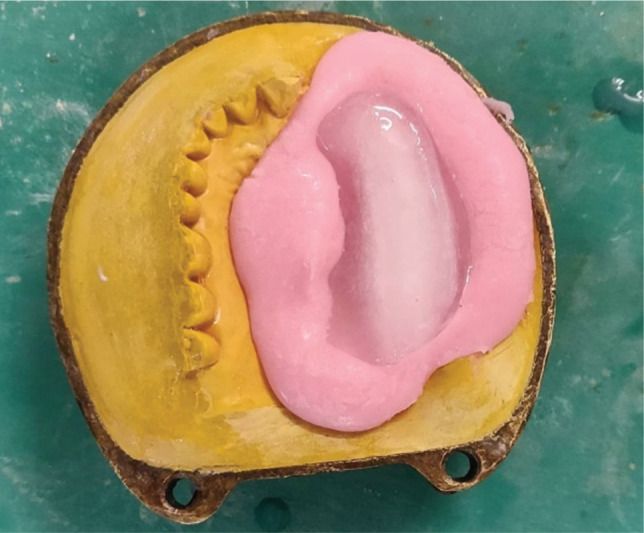
Ice block positioned on the base flask at the time of packing.

After setting the ice, the wax pattern was removed to get the ice block. (Figure 7). The heat cure acrylic resin (DPI) was mixed in dough consistency as per the manufacturer's instruction and was placed in a base and counter flask with an ice block interposed between them in the defect area (Figure 8). The mold was closed and clamped. The clamped mold was then immediately placed in an acrylizer without bench curing and was cured with a short curing cycle for 90 min at 74 °C followed by boiling at 100°C for 60 minutes.

Once the curing was complete, the flask was allowed to bench cool for an hour. Then, it was opened, and the prosthesis was retrieved. A small hole was drilled in the prosthesis for the retrieval of water to make it hollow. Water effortlessly came out of the prosthesis, and then the hole was closed with the self-cure acrylic resin (DPI).

**Figure 9:**
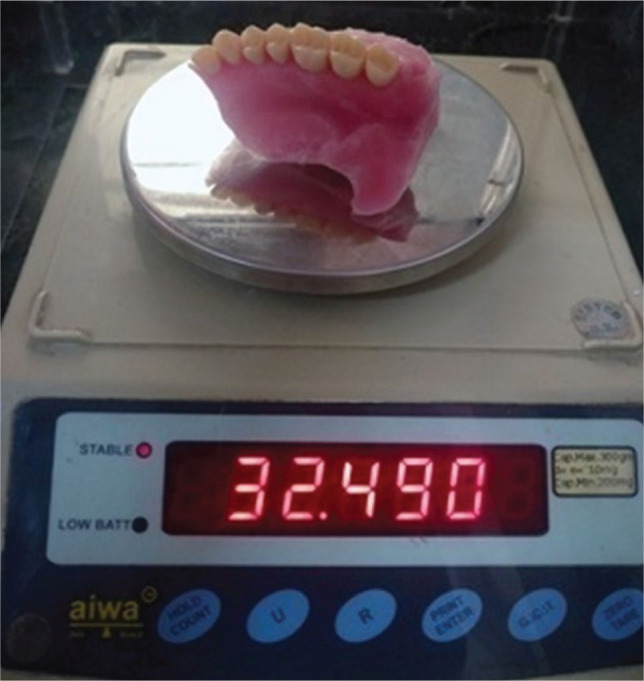
Weight of the prosthesis before hollowing of the prosthesis

**Figure 10:**
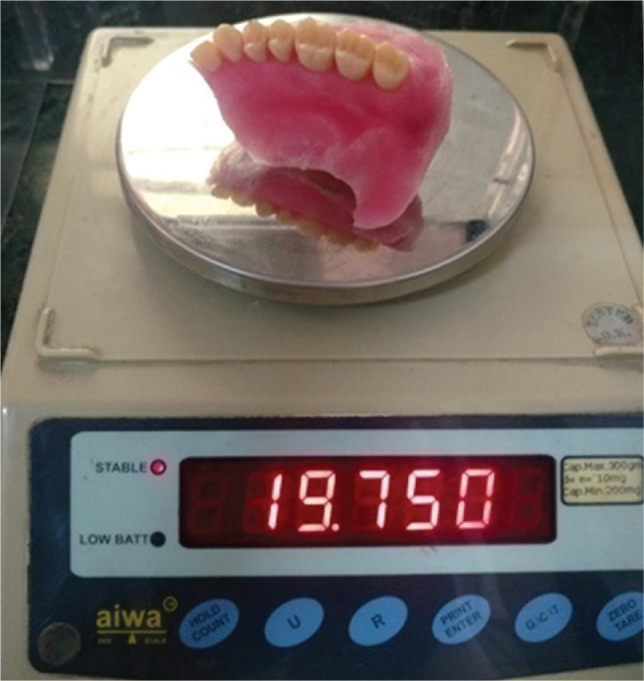
Weight of the prosthesis after prosthesis hollowing.

**Figure 11:**
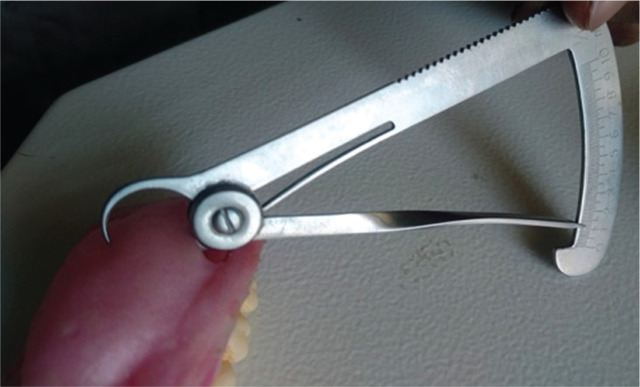
Uniform thickness of the prosthesis measured using a vernier caliper.

The obturator prosthesis was weighted before and after hollowing (Figures 9 and 10). Uniformity in the prosthesis thickness was achieved and measured with the help of a vernier caliper (Figure 11).

## DISCUSSION

While restoring a large defect, reducing the weight of the prosthesis by making it hollow is beneficial, especially for obturator prosthesis, wherein the heavyweight of the acrylic resin threatens the retention, stability, and support of the prosthesis, making the patient feel uncomfortable. Moreover, the heavyweight of the prosthesis increases the stress over the residual alveolar ridge and supporting bone that leads to further resorption of the denture-bearing foundation [18].

Several techniques and materials for the hollowing of the obturator prosthesis have been described in the literature to fabricate a lightweight prosthesis [10–16]. Materials such as silicon [19], a slurry of plaster and pumice [20], gelatin [21], modeling clay [22], cellophane wrapped in asbestos [23], salt [24], polyurethane foam [25], acrylic resin shim [26], have been used for hollowing the pros-thesis. However, these materials encountered problems during removal after processing through the small holes drilled in the prosthesis resulting in the heavyweight prosthesis. Moreover, some materials were costly and could not maintain the even thickness of the hollow portion, could not withstand the compression molding pressure, and could not sustains the curing temperature. Some materials increased the risk of seepage and discolored the prosthesis. Some material reacted with heat-cured acrylic resin resulting in porosities that affected the mechanical properties (Table 1). This article described a simple, unique, and less time-consuming technique to fabricate a hollow obturator prosthesis using ice.

**Table 1:** Pros and cons of various hollowing materials.

Sr.No	Material used for hollowing	Pros	Cons
**1**	Wax shim	• Ease of retrievability	• The difficulty of reliably seating wax-bolus in polymerizing resin during packing • Uniformity of resin thickness is difficult to achieve • Chances for dimensional change in the wax-bolus resulting from curing temperature • Time-consuming • Technique sensitive • Discoloration of final prosthesis occurs due to wax.
**2**	Sugar	• Highly soluble • Ease of removal • Moldable	• Roughness is observed on the hollow surface • Chance to break during compression molding • Strength is affected by porosities • Brittle
**3**			
**4**	Acrylic resin shim	• It leaves no lines of demarcation to discolor the denture • The technique is easy to carry out	• Time-consuming • Technique sensitive
**5**	Polyurethane foam	• Recontoured easily • It sustains curing temperatures	• Complex • Time-consuming • Difficult to remove after curing • The even thickness of the hollow portion is difficult to maintain
**6**	Plaster matrix	• Maintains the uniform thickness	• Chance to break during compression molding • Complex • Time-consuming • Brittle
**7**	Cellophane-wrapped asbestos	• Maintains the uniform thickness	• Allergy to asbestos • Difficult to remove
**8**	Silicone putty	• No difficulty in gauging resin thickness • Does not adhere to acrylic resin	• Difficult to remove • Time-consuming
**9**	Light-body coated gauze	• Simple technique	• Difficult to remove • Difficulty in gauging resin thickness • Compromises the strength of the prosthesis
**10**	Thermocol	• Simple to execute	• Gauging resin thickness is difficult • Left in the denture so no complete hollowing is achieved • Get displaced during compression molding
**11**	Dental stone	• Ease of gauging resin thickness	• Complex • Time-consuming • Difficult to remove
**12**	Play dough	• Maintains the uniformity of resin thickness	• Complex • Time-consuming • Difficult to remove
**13**	Salt	• Simple technique • Cost effective • No residual crystals left after curing	• Brittle • Chance to break during compression molding • The thickness of the hollow part cannot be kept uniform • Salt may react with heat cured acrylic resin and may lead to porosity
**14**	Gelatin soap	• Recontoured easily • It sustains curing temperatures • Easy retrievability	• Retrieval is done with hot water sprayed through the opening using a disposable syringe, resulting in the possibility of incomplete gelatin removal • Junction is formed between the two previously polymerized portions of the denture which may be at an increased risk of fluid seepage into the denture cavity.
**15**	Alum	• Ease of retrieval	• Leaving cellophane in the prosthesis • Difficulty in shaping the alum crystals to the defect

In 1978, Schneider *et al*. also used ice to hollow the obturator prosthesis. The authors advocated for the use of crushed ice in the defect area for hollowing. However, crushed ice can not completely fill the defect space and will result in uneven thickness of the hollow bulb, resulting in a further increase in the weight of the prosthesis [27]. To overcome this problem, the technique of using an ice block to precisely occupy the defect area and to achieve even thickness of the obturator prosthesis was developed.

The prosthesis weighed 32.49 grams before and 19.75 grams t after retrieval of water. A reduction of 12.74 grams of weight after retrieval of water is remarkable, which is achieved by the hollowing of the prosthesis. The uniform thickness of the prosthesis, of around 1.3 mm, was measured with a vernier caliper. Hollowing the prosthesis affects the flexural strength. If the clinician is more concerned about reducing the weight of the prosthesis, then the strength will be compromised, affecting the mechanical properties of the material [28].

When the boiling process is held in the pressure vessel, the water starts to boil at a higher temperature than 100°C. At a certain point, the energy absorbed by the water molecules makes them vibrate fast, and it attempts to escape and evaporate. However, the atmosphere around them exerts pressure on and thus limits the number of water molecules that can escape. Therefore, a minimal volume of water will expand, but the closed flask will limit it and not affect the accuracy of the obturator prosthesis.

### Pros of the present technique

- Ease of retrievability;
- Maintains even thickness of the hollow portion;
- Ice does not adhere to acrylic resin;
- The ice block can withstand the pressure of compression molding technique;
- Ice does not create porosities, so it does not compromise the strength of the prosthesis;
- Ice leaves no lines of demarcation to discolor the denture;
- Ice aids in the complete curing of the bulb portion;
- Total removal of ice is possible so complete hollowing of a prosthesis can be achieved;
- The procedure is not technique sensitive;
- Ease of availability;
- Inexpensive.

### Limitations of the present technique

- The flexural strength of the hollowed structure was not determined.

Other prostheses such as finger prosthesis, vaginal stent, complete dentures for severely atrophic ridges, and prosthesis for mandibular resection can also make use of ice to make them hollow. Future studies are suggested to evaluate the changes in the properties of the heat cure acrylic resin after processing with this technique. An experimental test program is recommended using numerical modeling as a tool to predict the behavior of the hollowed structure in terms of its mechanical properties [29]. Also, there is a need to perform uncertainty analysis with a sensitivity analysis toolbox consisting of Matlab functions that offer utilities for quantifying the influence of uncertain input parameters on uncertain model outputs [30].

## CONCLUSION

An alternative, simple, economical, time-saving, and predictable approach for the hollowing of the prosthesis has been described in this article. This technique overcomes the disadvantages of the older techniques. The ice block has the advantage of retrievability ease, and it does not adhere to acrylic resin. It also provides an even thickness of acrylic resin during the packing stage and resists deformation during the closure of the mold.
